# Comparison of different ionic liquids pretreatment for barley straw enzymatic saccharification

**DOI:** 10.1007/s13205-013-0157-x

**Published:** 2013-07-31

**Authors:** Sohrab Haghighi Mood, Amir Hossein Golfeshan, Meisam Tabatabaei, Saeed Abbasalizadeh, Mehdi Ardjmand

**Affiliations:** 1 Department of Chemical Engineering, South Tehran Branch, Islamic Azad University, Tehran, Iran; 2 Biofuel Research Team (BRTeam), Microbial Biotechnology and Biosafety Department, Agricultural Biotechnology Research Institute of Iran (ABRII), Mahdasht Road, Fahmideh Blvd., P.O. Box: 31535-1897, Karaj, Iran

**Keywords:** Ionic liquid, Pretreatment, Barely straw, Crystallinity index, Cellulose digestibility

## Abstract

Recently, application of ionic liquids due to their special solvency properties as a promising method of pretreatment for lignocellulosic biomass has received much attention. Chemical stability, temperature stability, non-flammability, low vapor pressure, wide liquidus range, and non-toxicity are among those unique properties. These solvents are also known as green solvents due to non-toxicity and low vapor pressure. The present study was set to compare the effect of five different ionic liquids namely, 1-ethyl-3-methyl imidazolium acetate, 1-ethyl-3-methyl imidazolium diethyl phosphate, 1-butyl-3-methyl imidazolium chlorides, 1,3-dimethyl imidazolium dimethyl phosphate, and 1-butyl-3-methylimidazolium-trifluoromethane sulfonate on barley straw in bioethanol production process. The performance of ionic liquids was evaluated based on the change observed in chemical structure, crystallinity index, and cellulose digestibility. Overall, 1-ethyl-3-methyl imidazolium acetate was found most effective in pretreating barely straw for bioethanol production. To the best of our knowledge, the present study reports different ionic liquids; some for the first time, for barely straw pretreatment.

## Introduction

Lignocellulosic biomass is suitable sources for conversion to bioethanol for they are abundant and cheap. However, conversion of lignocelluloses to bioethanol is faced with physical and chemical barriers. More specifically, crystalline structure of cellulose, presence of lignin, and covalent cross-linkages between lignin and hemicelluloses in cell wall obstruct the decomposition process of lignocellulosic materials (Haghighi Mood et al. 2013). Therefore, the goal of pretreatment is defined to overcome these obstacles including breaking down lignin structure, disrupting the crystalline structure of cellulose and cross-linked matrix of lignin and hemicelluloses, and increasing the porosity and surface area of cellulose (Alvira et al. 2010; Li et al. 2010).

To date, several pretreatment methods have been introduced including physical pretreatment (e.g. grinding and milling, microwave, and extrusion), chemical pretreatment (e.g. alkali, acid, organosolv, ozonolysis, and ionic liquid), physico-chemical pretreatment (such as steam explosion, liquid hot water, ammonia fiber explosion, wet oxidation, and CO_2_ explosion), and biological pretreatment. Most of the conventional pretreatment methods suffer from one or more drawbacks. For instance, dilute acid pretreatment needs costly corrosion resistant equipments and besides, leads to the production of a significant amount of fermentation inhibitors during the process (Yoon et al. 2011). Biological pretreatment also requires large space and is lengthy. Moreover, it needs continuous monitoring of microorganism growth (Wyman et al. 2005; Chandra et al. 2007). As for the organic solvents, flammability and explosion are of major concern (Galbe and Zacchi 2007). Ammonia fiber explosion, hot water, and steam explosion processes are also costly and are not yet economically feasible due to high operation cost basically due to the high cost of ammonia and being energy-intensive, respectively (Guragain et al. 2011). Mechanical pretreatment is energy intensive and costly as well (Haghighi Mood et al. 2013).

Recent attempts have been striving to make pretreatment methods more efficient, environmentally friendly, and cost effective (Li et al. 2009). Among them, application of ionic liquids (ILs) as a promising pretreatment method has gained much attention. ILs are organic salts composed of anions and cations and melt below 100 °C (Tan et al. 2010). The mild process condition and unique safety feature of the chemicals used in this method are regarded as the main advantages. ILs remain liquid in a wide range of temperature. Moreover, they have low vapor pressure and high chemical and thermal stability. As a result of these unique features, they are known as green solvents (Tan et al. 2010).

Depending on the selection of cations and anions to be involved in the structure of ILs, their properties (i.e. viscosity, melting point, and polarity) could be tuned (Mora-Pale et al. 2011). Depending on the type of ILs, they are capable of dissolving carbohydrates and lignin. In fact, hydrogen bonds are formed between the non-hydrated ions of ILs and the sugar hydroxyl protons and as a result, the complex network of cellulosic biomass polymers, hemicelluloses, and lignin is broken down (Alvira et al. 2010). The regenerated cellulose has more amorphous and porous structure than those in untreated lignocellulosic biomass. Therefore, regenerated cellulose is much more susceptible to enzymatic hydrolysis (Zhao et al. 2009). Moreover, one of the most important advantages of ILs solvents is their recyclability. More specifically, these solvent can be reused (recycled) without affecting their performance in dissolution of cellulose (Li et al. 2009).

In this study, barley straw was pretreated using five different ILs, i.e. 1-ethyl-3-methyl imidazolium acetate ([EMIM][AC]), 1-ethyl-3-methyl imidazolium diethyl phosphate ([EMIM][DEP]), 1-butyl-3-methyl imidazolium chlorides ([BMIM][CL]), 1,3-dimethyl imidazolium dimethyl phosphate ([MMIM][DMP]), and 1-butyl-3-methylimidazolium-trifluoromethane sulfonate ([BMIM][OTf]). The performance of ILs was evaluated based on the change observed in chemical structure of the biomass, cellulose crystallinity index, and cellulose digestibility.

## Materials and methods

### Materials and preparation

Barley straw samples were collected from the research farm of Seed and Plant Improvement Institute. The straws were dried under sun before shredded into pieces. Then, the shredded straws were sieved to obtain fractions with a particle size of 0.420 mm and their composition, i.e. cellulose, hemicellulose, and lignin contents was determined based on National Renewable Energy Laboratory (NREL/TP-510-42618) (Sluiter et al. 2008). The ILs 1-ethyl-3-methyl imidazolium acetate, 1-ethyl-3-methyl imidazolium diethyl phosphate, 1-butyl-3-methyl imidazolium chlorides, 1,3-dimethyl imidazolium dimethyl phosphate and 1-butyl-3-methylimidazolium-trifluoromethane sulfonate were purchased from Sigma-Aldrich. Cellulase from *Trichoderma reesei* ATCC 26921 and Cellobiase from *Aspergillus niger* were purchased from Sigma-Aldrich as well. The other chemicals used in this study included sulfuric acid 95–97 % (Fluka), citric acid monohydrate (Sigma), sodium hydroxide ≥97 % (Sigma-Aldrich), 3,5-dinitrosalicylic acid 98 % (Aldrich), hydrochloric acid 37 % (Merck), potassium sodium tartrate tetrahydrate (Merck), sodium metabisulfite ≥99 % (Sigma-Aldrich), Tetracycline (Sigma), and cycloheximide ≥93 % (Fluka).

### Barley straw pretreatment

A 4 % (w/w) barley straw solution or in other words, a 96 % IL solution was prepared by combining 200 mg of barley straw with 4.8 g IL in a test tube. The test tubes containing the samples were stirred (150 rpm) and heated in an oil bath at 110 °C for 90 min. All experiments were carried out in triplicates. After 90 min of incubation, the reaction mixtures were cooled down to 60 °C and then 50 ml deionized water as an anti solvent was added to precipitate and regenerate the dissolved cellulose. Next, the precipitated material was filtered through filtering paper (Whatman No. 2) using Buchner funnel under a reduced pressure and washed with deionized water in order to ensure that excess ionic liquid had been removed. Then prior to enzymatic hydrolysis, the precipitates were dried at 60 °C for 48 h and their composition was determined as mentioned earlier.

### Enzymatic hydrolysis

Enzymatic saccharification of pretreated and untreated barley straw was carried out at 50 °C and 150 rpm in a shaker incubator. The cellulase activity was determined based on NERL. Cellulase and β-glucosidase were loaded in at 40 FPU g^−1^ substrate and 200 CBU g^−1^ substrate, respectively. Samples were withdrawn at 3, 6, 12, 24 and 72 h for analysis. For each vial, 5.0 ml sodium citrate buffer 0.1 M (PH 4.8) was added to the equivalent amount of 0.15 g total barley straw biomass. Moreover, 40 μL (400 μg) tetracycline and 30 μL (300 μg) cycloheximide were also added into each vial to prevent the growth of organisms during the digestion. After addition of the enzymes, the volume of each vial was brought to 10 ml by addition of deionized water. Glucose concentration in each vial was determined by high-performance liquid chromatography (HPLC) with an RI detector (Knauer, Germany) equipped with a Eurokat H carbohydrate analysis column (Knauer, Germany). The mobile phase was acidified waster (0.01 N sulfuric acid, pH 2,), at a flow rate of 1 ml min^−1^ with a column temperature of 65 °C.

### FTIR analysis

The chemical structure of untreated and pretreated barley straw was characterized using Fourier transform infrared (FTIR-FIR) spectrometry (Equinox 55, Bruker Germany). All biomass samples were dried and mixed with potassium Bromide (KBr) before pressing the sample into discs.

### Scanning electron microscopy

The effect of pretreatment on the morphology of the barley straw was observed with scanning electron microscopy (SEM) (TESCAN_VEGA) at an acceleration voltage of 20 kV. Samples were mounted on aluminum sample stubs and sputtered with a thin layer of gold. Finally, many spots (at least five) were considered for each sample under different magnifications.

### Crystallinity measurement

X-ray powder diffraction (XRD) (Siemens, Model D5000, Germany) was applied to characterize the crystallinity of lignocellulosic materials for pretreated and untreated barley straw. XRD data were measured at 25 °C using a Fe tube (voltage 35 kW, 25 mA). Samples were scanned over the range of 5°–70° with a step size of 0.02 s and step time of 10 s. Crystallinity index (Crl) was determined based on the XRD data and calculated using the following formula (Segal et al. 1959):

In which, *I*_002_ is the intensity for crystalline portion of biomass at about 2*θ* = 22.5 and *I*_am_ is the peak for the amorphous portion (i.e., cellulose, hemicelluloses and lignin) at about 2*θ* = 16.6. The second highest peak after 2*θ* = 22.5 was 2*θ* = 16.6, and was assumed to correspond to amorphous region (Kumar et al. 2009).

## Result and discussion

The application of ILs has been received much attention recently. ILs as solvents possess some advantages such as no cellulose decomposition, easy processing, easy cellulose regeneration (precipitation upon addition of anti-solvent, e.g. deionized water), and no toxicity (Tam-Anh et al. 2010). In the present study, a wide range of ionic liquids, i.e. 1-ethyl-3-methyl imidazolium acetate, 1-ethyl-3-methyl imidazolium diethyl phosphate, 1-butyl-3-methyl imidazolium chlorides, 1,3-dimethyl imidazolium dimethyl phosphate, and 1-butyl-3-methylimidazolium-trifluoromethane sulfonate were examined in order to find the best solvent for barley straw at 110 °C for 90 min. The composition of barely biomass, i.e. cellulose, hemicellulose, and lignin, was 40.8, 21.76 and 12.18 %, respectively. Changes in the composition of the solid phase promoted by ILs pretreatment were evaluated by measuring cellulose content. The cellulose content after pretreatment by [EMIM][AC], [EMIM][DEP], [MMIM][DMP], [BMIM][CL], and [BMIM][OTf] was recorded at 51.83, 43.10, 42.75, 42.49 and 40.18, respectively. As clearly observed [EMIM][AC] led to the highest compositional change in the barely biomass. These differences could be attributed to the important ionic parameters such as cation and anion size as well as hydrogen bond basicity (Mäki-Arvela et al. 2010). In the present study, [EMIM]^+^ was found of better dissolution capability in comparison with [BMIM]^+^. This could be explained by the fact that small cations ([EMIM]) are often more efficient in dissolving cellulose than larger ones ([BMIM)] (Kosan et al. 2008). Moreover, the better performance of [MMIM][DMP] compared to [BMIM][CL] and [BMIM][OTf] was due to its higher basicity (Mäki-Arvela et al. 2010).

### Cellulose digestibility

After pretreatment and 3 h hydrolysis, the lowest glucose release was obtained for the untreated barley straw while the highest (1.37 mg glucose ml^−1^) was attributed to the barley straw pretreated by [EMIM][AC]. At the end of the experiment at 72 h after the commencement of the hydrolysis process, the highest concentration still belonged to barley straw pretreated by [EMIM] [AC] at 3.95 mg glucose ml^−1^. The results obtained mark [EMIM][AC] as the best IL pretreatment choice for significantly improving the enzymatic saccharification in comparison with the other four ILs used.

**Fig. 1:**
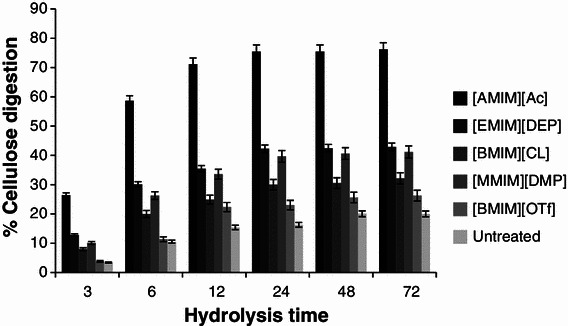
Comparison of untreated (control) and treated barely samples with different ionic liquids in terms of cellulose digestion (%) versus time

Based on the enzymatic hydrolysis data, cellulose digestibility was calculated as described by NREL/TP-510-42629 (Selig et al. 2008). Figure 1 shows cellulose digestibility profiles for untreated barley straw as well as barley straw samples pretreated by five ILs while the same enzyme loading was applied to all samples during the hydrolysis process. Significantly higher saccharification was achieved using [EMIM][Ac]-pretreated barley straw showed with cellulose digestibility reaching 76 % within 72 h, whereas digestibility of untreated barley straw only reached 20 %.

This difference caused by ILs in particular [EMIM][Ac] could be ascribed to the loss of intra- and inter-molecular hydrogen bonds leading to the formation of amorphous cellulose and consequently increased surface area. The latter leads to better enzyme accessibility and increased binding sites in recovered cellulose fibers (Li et al. 2010).

### FTIR analysis

**Fig. 2:**
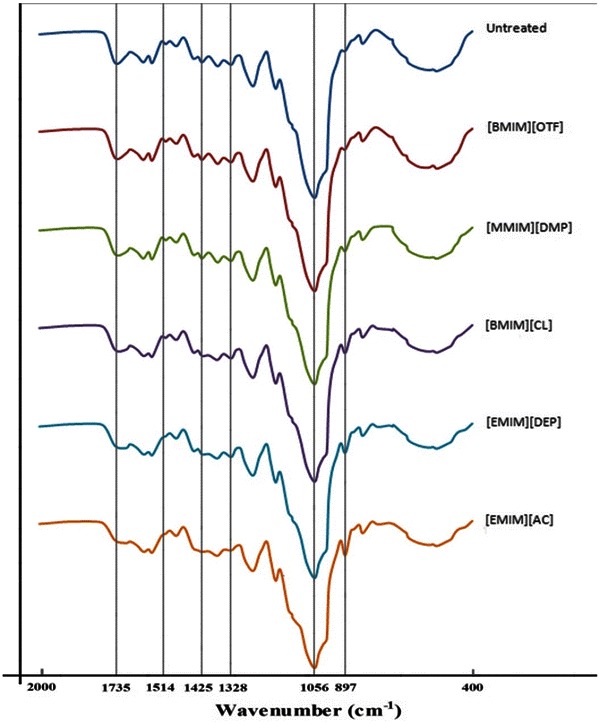
The FTIR spectra of barley samples pretreated by different ionic liquids

The chemical structure of untreated and pretreated barley straw samples was analyzed using FTIR. As shown in Fig. 2 and Table 1, the spectra generated for samples pretreated by ILs were similar to that of the untreated barley straw; however, there were some small differences observed. For instance, at 897 cm^−1^, the peak obtained was more intense in cases of [EMIM][AC]- and [EMIM][DEP]-pretreated barley straws compared with untreated, [BMIM][CL]-, [MMIM][DMP]-, and [BMIM][OTf]-pretreated barley straw. In the presence of amorphous cellulose, the band at 897 cm^−1^, which characterizes the C–O–C stretching at β-1,4-glycosidic linkage, is strong and sharp. The peak at 1,430 cm^−1^ can be assigned to bending vibration of CH_2_. This band is strong in crystalline cellulose and weak in amorphous cellulose. So, the crystalline cellulose in treated samples by [BMIM][CL], [MMIM][DMP], [BMIM][OTf] and untreated barley straw is more than the samples treated by [EMIM][AC] and [EMIM][DEP].

**Table 1:** FTIR intensity values obtained for barley samples pretreated by different ionic liquids

Treatment	FTIR peaks (cm^−1^)					
	897	1,056	1,328	1,425	1,514	1,735
Untreated	91.020	96.810	91.557	91.230	90.652	91.494
[BMIM][OTF]	91.054	96.630	91.554	91.231	90.655	91.420
[MMIM][DMP]	91.155	96.425	91.556	91.232	90.652	91.302
[BMIM][CL]	91.343	96.404	91.574	91.240	90.707	91.436
[EMIM][DEP]	91.405	96.323	91.564	91.247	90.838	91.127
[EMIM][AC]	91.590	96.026	91.388	91.251	90.848	91.033

The results obtained indicate that untreated barley straw contained higher amount of crystalline cellulose. On the other hand, cellulose in barley straw became more amorphous after pretreatment using ILs. It could be concluded that the amount of amorphous cellulose was highest in the barely sample pretreated by [EMIM][AC], followed by [EMIM][DEP], [BMIM][CL], [MMIM][DMP], and [BMIM][OTf], respectively. The peaks at 1,328 and 1,514 cm^−1^ were indicators of lignin characteristic. More specifically, 1,328-cm^−1^ peak reveals the aromatic hydroxyl groups generated by the cleavage of ether bonds within lignin whereas that of 1,514 cm^−1^ is associated with the aromatic skeletal modes of lignin (Hsu et al. 2010).

As observed in Fig. 2 and Table 1, barley straw samples subjected to IL pretreatment were delignified slightly for the peaks generated at 1,328 and 1,514 cm^−1^ were identical and that there was a subtle difference between the ILs pretreated samples and the untreated one. However, barley straw subjected to [EMIM][DEP] pretreatment was delignified slightly more efficiently in comparison with the other IL pretreatments. Overall as could be concluded from Fig. 2 and Table 1, using ILs is not a suitable method for removing lignin.

### Scanning electron microscopy

**Fig. 3:**
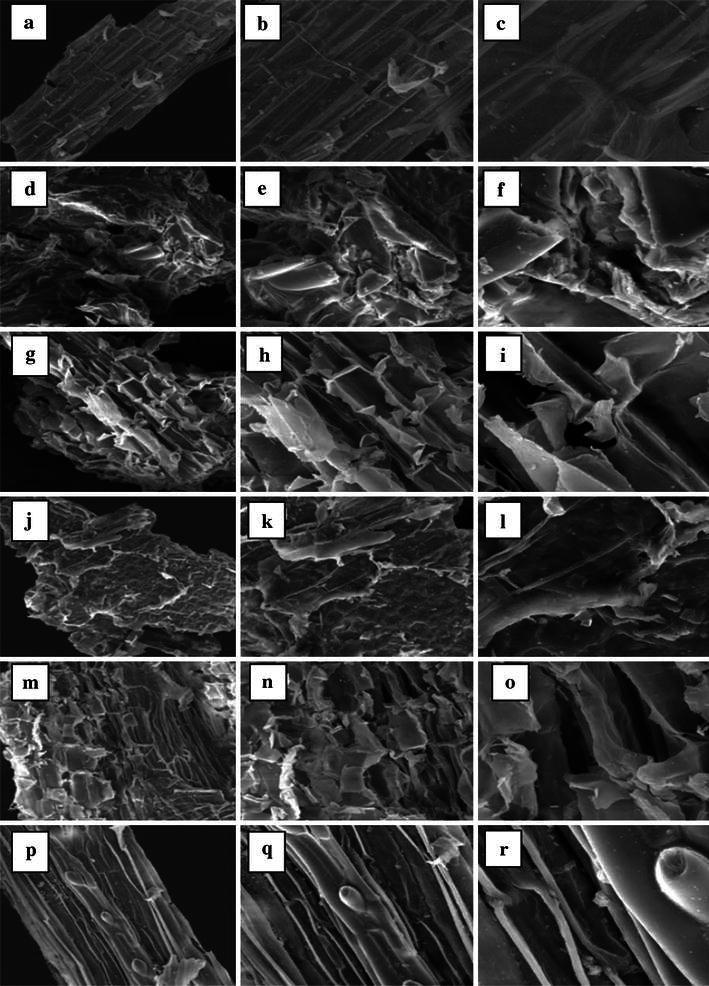
SEM images of barley straw; **a**–**c** raw barley straw (×500, ×1,000, ×3,000); **d**–**f** [EMIM][AC]-pretreated barley straw (×500, ×1,000, ×3,000); **g**–**i** [EMIM][DEP]-pretreated barley straw (×500, ×1,000, ×3,000); **j**–**l** [BMIM][CL]-pretreated barley straw (×500, ×1,000, ×3,000); **m**–**o** [MMIM][DMP]-pretreated barley straw (×500, ×1,000, ×3,000); **p**–**r** [BMIM][OTf] pretreated barley straw (×500, ×1,000, ×3,000)

Figure 3 presents the physical structural changes obtained in barley straw during the ILs pretreatment. SEM images of untreated and ILs-pretreated barley straw samples were taken at 500×, 1,000× and 3,000× magnifications. The results obtained indicate that the untreated barley straw had a highly fibrillar and intact morphology (Fig. 3a–c) in comparison with those that underwent IL-pretreatments (Fig. 3d–r). Among the ILs used, [EMIM][AC] pretreatment was clearly proven to have altered the structure of barley straw the most (Fig. 3d–f). As shown, the surface has become swollen and loose and the original fibrous structure has been completely distorted after the pretreatment by [EMIM][AC]. In other words, the fibrous structure of the barely straw has been transformed into a porous and amorphous form after the [EMIM][AC] pretreatment.

[EMIM][DEP] and [MMIM][DMP] pretreatments had similar effects on barley straw (Fig. 3g–l) and led to maximum alterations in barley straw structure after [EMIM][AC] pretreatment. On the ILs list, the less effect on barley straw physical structure belonged to [BMIM][OTf] pretreatment which was incapable of making any significant alterations (Fig. 3p–r).

### Barley straw crystallinity

Biomass crystallinity is an important feature affecting enzymatic hydrolysis. Different pretreatment methods can alter cellulose crystal structures by disrupting inter- and intra-chain hydrogen bonding of cellulose fibrils (Mosier et al. 2005). In this study, the features of regenerated barley straw samples after various IL pretreatments were examined using X-ray diffraction and were also compared to those of untreated barley straw. Untreated barley straw was found highly crystalline (59.5 Crl). After [EMIM][AC] pretreatment, Crl index of barley straw was decreased significantly to 15.2 revealing minimal structural order in cellulose after the pretreatment. This Crl value was the least when compared with those achieved through the application of the other ILs in the pretreatment process. In other words, this sharp decrease in crystallinity due to the [EMIM][AC] pretreatment confirms that the regenerated products were highly amorphous and thus, cellulose surface accessibility and consequently the efficiency of enzymatic hydrolysis were considerably increased. After [EMIM][DEP], [BMIM][CL], [MMIM][DMP], and [BMIM][OTf] pretreatments, Crl value of barley straw was decreased to 30, 48, 30.5, and 57, respectively.

## Conclusion

Due to the worldwide cultivation of barley, its straw is one of the most important feedstock for the production of fermentable sugar and bioethanol. Among the 5 different ILs examined, [EMIM][AC] was found to have led to the highest degree of highest cellulose conversion. The SEM, FTIR, and XRD analyses ranked [EMIM][AC] pretreatment followed by [EMIM][DEP] pretreatment as most efficient in terms of altering the physical structure of barley straw. Overall, [EMIM][Ac]-pretreated barley straw showed significantly higher saccharification with cellulose digestibility reaching 76 % after 72 h, whereas digestibility of untreated barley straw only reached 20 %.
